# Evaluation of the Combined Treatment Approach “Robin” (Standardized Manual and Smartphone App) for Adolescents at Clinical High Risk for Psychosis

**DOI:** 10.3389/fpsyt.2019.00384

**Published:** 2019-06-06

**Authors:** Nina Traber-Walker, Miriam Gerstenberg, Sibylle Metzler, Maria Raquel Joris, Markus Karr, Nadja Studer, Marina Zulauf Logoz, Alexander Roth, Wulf Rössler, Susanne Walitza, Maurizia Franscini

**Affiliations:** ^1^Department of Child and Adolescent Psychiatry and Psychotherapy, University Hospital of Psychiatry, University of Zurich, Zurich, Switzerland; ^2^The Zurich Program for Sustainable Development of Mental Health Services (ZInEP), University Hospital of Psychiatry Zurich, Zurich, Switzerland; ^3^Department of Psychiatry, Psychotherapy and Psychosomatics, Zürich University Hospital of Psychiatry, Zurich, Switzerland; ^4^Department of Psychiatry and Psychotherapy, Charité University Medicine, Berlin, Germany; ^5^Laboratory of Neuroscience, LIM27, Institute of Psychiatry, University of Sao Paulo, São Paulo, Brazil; ^6^Neuroscience Center Zurich, University of Zurich and ETH Zurich, Zurich, Switzerland; ^7^Zurich Center for Integrative Human Physiology, University of Zurich, Zurich, Switzerland

**Keywords:** psychosis, eMental health, psychological intervention, youth mental health, clinical high risk of psychosis, early intervention

## Abstract

**Introduction:** The prevention of schizophrenia and other psychotic disorders has led researchers to focus on early identification of individuals at clinical high risk (CHR) for psychosis and to treat the at-risk symptoms in the pre-psychotic period. Although at-risk symptoms such as attenuated hallucinations or delusions are common in adolescents and associated with a marked reduction in global functioning, the evidence base of effective interventions for adolescents at CHR state and even ﬁrst-episode psychosis is limited. Thus, the present protocol describes a study design that combines therapy modules for CHR adolescents with a smartphone application supporting the young individuals between the therapy sessions. The treatment approach “Robin” is based on existing therapy strategies for adolescents with first episode of psychosis and the available recommendations for adults with at-risk symptoms.

**Methods:** The evaluation aims firstly to compare the efficacy of Robin in 30 CHR adolescents aged 14–18 to an active control group (treatment as usual) from a previous study. Primary outcome measures will be at-risk symptomatology, comorbid diagnosis, functioning, self-efficacy, and quality of life. For the prospective intervention condition (16 weekly individual sessions + a minimum 4 family sessions), help-seeking adolescents with CHR for psychosis, aged 14–18, will be recruited over 3 years. At-risk and comorbid symptoms, functioning, self-efficacy, and quality of life are monitored at six time points (baseline, during the treatment period; immediately after intervention; and 6, 12, and 24 months later) and compared with the respective measures of the active control group.

**Discussion:** To the best of our knowledge, this is the first controlled trial to test the efficacy of a specific early psychosis treatment in combination with a smartphone application for adolescents at CHR for developing psychosis. The results of the study are expected to add information that may substantially decrease the burden of CHR adolescents and increase their resilience. It may offer age-adapted and targeted strategies to guide clinicians in the treatment of these vulnerable individuals. Furthermore, research in the field of early intervention will be enriched by our findings.

**Clinical Trial Registration:** www.ClinicalTrials.gov, identifier NCT03829527.

## Introduction

Psychotic disorders are severe mental illnesses that typically emerge in late adolescence or early adulthood (1). The disorders lead to considerable social and economic deficits, as well as burden for the individual and family (2). Prior to the onset of the first psychotic episode, the majority of the individuals experience attenuated psychotic symptoms, known as at-risk symptoms (3).

Two different sets of clinical high risk (CHR) criteria have been developed for the purpose of early identification of individuals at CHR: 1) the basic symptom criteria focusing on subtle, subjectively experienced changes in cognition and perception and 2) the ultra-high-risk criteria, such as attenuated positive symptoms indicating the imminent manifestation of psychosis (4). In the present article, we use the term CHR state, which includes both of these current risk concepts.

There is an ongoing discussion as to whether or not to treat at-risk symptoms (5–7). The rates of CHR individuals transitioning to psychotic disorders have declined over the years of early detection research (8), and the specificity of at-risk symptoms is questioned (5, 9). At-risk symptoms are common in the general population, particularly during adolescence; it is a clinical challenge as to how to differentiate between young people who are at risk and those who are not (10, 11). Nevertheless, leading experts in the field of early intervention in psychosis have claimed that treatment of at-risk symptoms in the help-seeking population is justified independently of the risk of conversion to psychosis [for example, Refs. (12–15)].

CHR individuals and their caregivers report high levels of distress due to these symptoms (16). The CHR state is associated with suicidality, impairments to global functioning, and a decline in quality of life (17–23). CHR individuals report poorer social relationships and higher rates of loneliness than do healthy peers (24). Furthermore, CHR individuals seem to have poor coping skills, low levels of self-efficacy, and negative metacognitive belief similar to that seen in patients with depression or with a frank psychosis (25–27). Recent studies have shown a high prevalence of psychiatric comorbidities in CHR populations for psychosis (28, 29), especially in younger CHR individuals (20). Moreover, the CHR state is associated with long-term implications, independent of conversion to psychosis. A recent study showed that about half of non-transitioned CHR individuals remained functionally impaired and presented with at least one comorbid disorder at a 6-year follow-up (30). On the other hand, CHR individuals who convert to psychosis have shown poorer clinical outcomes than do first episode psychosis patients without a history of at-risk symptoms (31). Consequently, current data clearly favor targeting and treating at-risk symptoms in help-seeking individuals.

Although at-risk symptoms are common in adolescents and associated with a marked reduction in functioning in this age group (23), the evidence base required to guide effective interventions for CHR adolescents and even ﬁrst-episode psychosis is limited (15, 32–35). Most intervention studies in the CHR population have included adult patients, with only a few including younger patients (36–40). A majority of them used the same treatment approaches for adults and adolescents. Consequently, there is a lack of investigation targeting age-appropriate treatment strategies in this vulnerable age group. The transition from childhood to adulthood is a very complex developmental stage, and age-adapted intervention strategies are required. The CHR state can disrupt social and psychological development, such as the achievement of educational goals or the construction of peer relationships. Therefore, minor CHR individuals need treatment strategies that address the complex symptomatology, the associated burden, and their reduced functioning. Furthermore, when focusing on engagement in therapy during first psychotic episodes, the data demonstrated that age-appropriate treatment approaches drawing from youth specific interests are needed to address and involve the younger patients (32, 41).

To fill this gap, the experts from the specialized outpatient care unit for early intervention in psychosis at the Department of Child and Adolescent Psychiatry and Psychotherapy, Psychiatric University Hospital of the University of Zurich (CAPS), developed the specialized treatment approach “Robin” for CHR adolescents. The treatment program Robin is an integrated psychotherapeutic approach. Robin comprises individual therapy, family sessions, and a smartphone application for supporting patients between sessions. The therapy modules are based on treatment strategies in CHR adults (6, 14, 42–44) and recommendations for adolescents with first episodes of psychosis (33, 45, 46). It follows the guidance on early intervention in CHR states of psychosis of the European Psychiatric Association (EPA) (15).

The treatment modules are also based on existing concepts of cognitive behavioral therapy (CBT). CBT is currently the most widely approved psychological intervention in early intervention of psychosis and recommended by EPA guidance (15). Previous studies showed that CBT may prevent or at least postpone a first psychotic episode in CHR individuals (47); however, these promising findings need replication, and studies applying CBT in adolescents are rare. Cognitive remediation is used as a supplement treatment and focuses on improving cognitive and social skills. Additionally, the treatment modules comprise aspects of systemic therapy, which have been found to be efficacious in the treatment of psychosis in adult patients (48). Assuming that family environment plays a more relevant role in adolescents than in adults, there is a strong rationale for the possible benefits of systemic intervention in adolescents with CHR state. The initial studies of systemic treatment approaches in adolescents with psychosis (32) and CHR adolescents are promising (36, 39).

In addition to the above, the intervention also includes the smartphone application “Robin Z.” Smartphones have become everyday devices, and most people use them in their daily routine. Previous analyses showed that for young people, in particular “digital natives,” it may be helpful to develop technology-based treatment approaches as a way of connecting with them about mental health issues (49–51). There is increasing interest in using mobile technologies such as smartphone applications in mental health care (52–54). Primarily, mobile technologies were utilized to collect research data. These technologies allow for real-time assessment. This provides more accurate data about real-world contexts in which experiences are made or behaviors occur (55, 56). So risk and protective factors in the psychosocial environment and their impact on mental health can be more easily identified. Further, studies with smartphone assessments showed higher engagement in completing all examinations and lower dropout rates than did studies with paper-and-pencil assessments (57, 58). Recently, research projects have examined feasibility and validity of mobile technologies in supporting therapy (53, 59). Smartphone interventions have many advantages like accessibility, portability, 24-h support, or real-time intervention. Using smartphones in everyday situations (i.e., on the bus, at school, and on the street) is routine, and so interventions *via* smartphone are not likely to be stigmatizing (49). The problem of recall bias in retrospective reports can be reduced or avoided. This helps the psychotherapist to give more tailored feedback to support and reinforce changes in the patients’ behavior according to their psychological health. Furthermore, self-monitoring also provides advantages for the patients themselves. The repeated measurement of their symptoms and mood can reveal insights into their psychological processes (60). Reid and colleagues (61) found an increased self-awareness regarding their symptoms and mood after using a symptom-monitoring smartphone application in a group of younger psychiatric patients. There is also evidence that medication adherence can be improved through smartphone intervention (62, 63).

In the treatment of psychotic disorders, the first research results from the use of smartphone applications are promising (62, 64–68); however, there is little known about mobile technologies addressing at-risk symptoms (50). Despite current analysis demonstrating young people would be interested in using mobile technologies, there is a lack of investigations with this population (49, 51). The available studies have shown a high acceptance and subjective satisfaction with mobile interventions in young people with psychiatric problems (57, 61, 69).

There is great heterogeneity across symptomology and functioning in the CHR population (30, 70). As a consequence, individualized adaptive treatment approaches are required. While some individuals will respond to an intervention, others will not. Robin is an individually tailored approach, and the treatment is personalized to the individuals’ special needs depending on their stage of illness, presence of comorbidities, or functional impairment.

The current clinical trial “Evaluation of the Treatment program Robin” (ETRo) has two main study goals:

1. to investigate the feasibility and impact of the combined treatment program: the structured therapy and the smartphone application; and
2. to investigate the efficacy of this specific intervention versus treatment as usual (TAU).

Hypotheses of the study:

Hypothesis 1: Robin is feasible and accepted in this young population of CHR individuals. The adolescents will show high treatment engagement.
Hypothesis 2: Robin improves the quality of life and self-efficacy at 6-, 12-, and 24‐month follow-up in individuals with CHR compared with individuals with CHR who received TAU.
Hypothesis 3: Robin enhances the rate of adolescents with clinical and psychosocial functional remission at 6-, 12-, and 24‐month follow-up than does TAU.

## Methods and Study Design

The ETRo Trial is a prospective, single-center, follow-up study comparing a specific treatment program for adolescents with at-risk symptoms with a sample from a previous study having received TAU.

### Participants

Participant recruitment will run between September 2017 and continue until September 2020 in the early recognition center for psychosis of the Department of Child and Adolescent Psychiatry and Psychotherapy, Psychiatric University Hospital of the University of Zurich (CAPS). The ETRo trial is a naturalistic treatment study. All participants will be help-seeking adolescents (aged 14–18) with at-risk symptoms assessed at the early recognition center. The purpose of the study, the study process, risks, benefits, and alternatives will be explained to all participants and their caregivers according to ethical principles. All participants will be asked to give a written informed consent in accordance with the national law. For inclusion, participants must meet at least one of the following at-risk criteria: 1) at least two self-experienced and self-reported cognitive basic symptoms as assessed by the Schizophrenia Proneness Interview Child and Youth Version (SPI-CY) (71) and/or 2) at least one attenuated psychotic symptom for psychosis assessed by the Structured Interview for Prodromal Syndromes (SIPS) (72).

Exclusion criteria for study participation include a diagnosis of a psychotic disorder, current substance or alcohol dependence, aged below 14, insufficient German or English language ability, or low intellectual abilities with IQ < 75.

### Study Design

**Figure 1:**
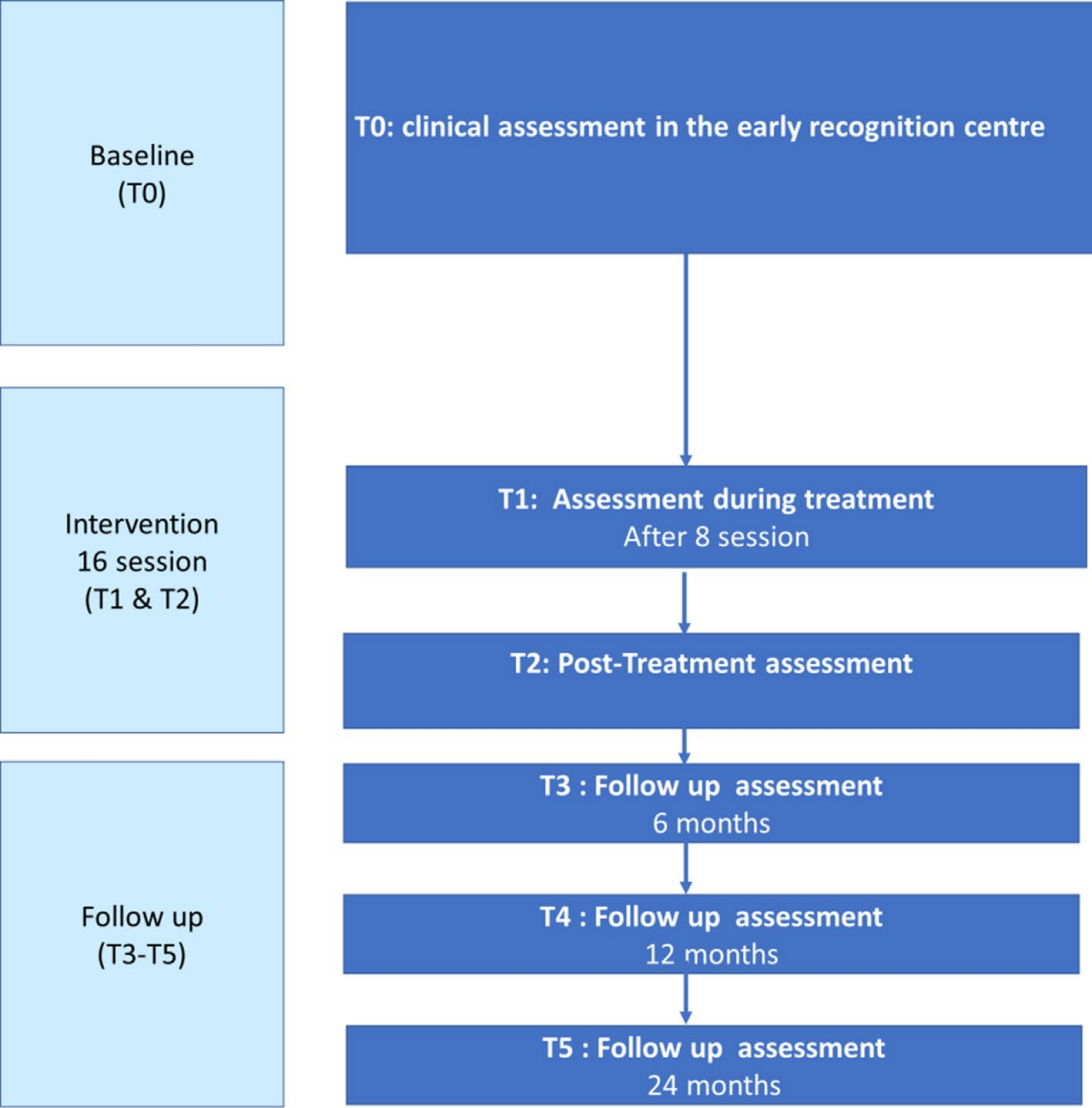
Flow chart of the assessments from baseline to 24 months of follow-up.

Within this prospective evaluation data for at-risk symptoms and comorbid symptoms, global functioning, self-efficacy, and quality of life will be collected at six time points (baseline, during the treatment period; immediately after intervention; and 6, 12, and 24 months later). The treatment period will be 16 weekly, individual sessions combined with at least four family sessions starting after the baseline clinical assessment (**Figure 1**).

#### Study Intervention

The integrated psychotherapeutic treatment approach Robin combines standardized treatment modules with a smartphone application. The treatment is targeted at at-risk symptoms as well as comorbid disorders. Improving quality of life and daily functioning is also a primary target in therapy and outcome measures. An important part of the program is the work on the individuals’ self-esteem and their experience of self-efficacy. The program is solution and resource oriented in reframing one’s symptoms. A variety of questioning techniques are used to enrich the individuals’ perspectives, to identify problems, and to create more possibilities in dealing with daily difficulties. Weekly homework tasks are usually given to transfer the insights and experiences from the therapy in the individuals’ daily life.

The first session is a family session directly after the clinical baseline assessment. In this session, there is a detailed psychoeducation to develop a concept of the CHR state with the adolescent and their caregivers. The therapeutic goals will be declared. The therapeutic modules provide information and individual approaches to target specific complaints of the adolescent within the following 15 sessions. The individuals are asked to make a daily record of their symptoms, mood, sleeping patterns, and alcohol and drug use over the entire treatment period. The records are discussed every week in the first part of the therapy session.

The psychotherapists consist of two female graduated psychologists, NT-W and SM, trained in research early recognition programs and experienced in treatment of clinical outpatients. MF, medical doctor, head of the early recognition service for adolescents, and the respective psychologist will conduct the family sessions together.

In accordance with the EPA guidance (15), low-dose second-generation antipsychotics are used when at-risk symptoms are severe and progressive with only minimal or clearly declining insight. The medication is monitored and supervised by M. F.

##### App Robin Z

**Figure 2:**
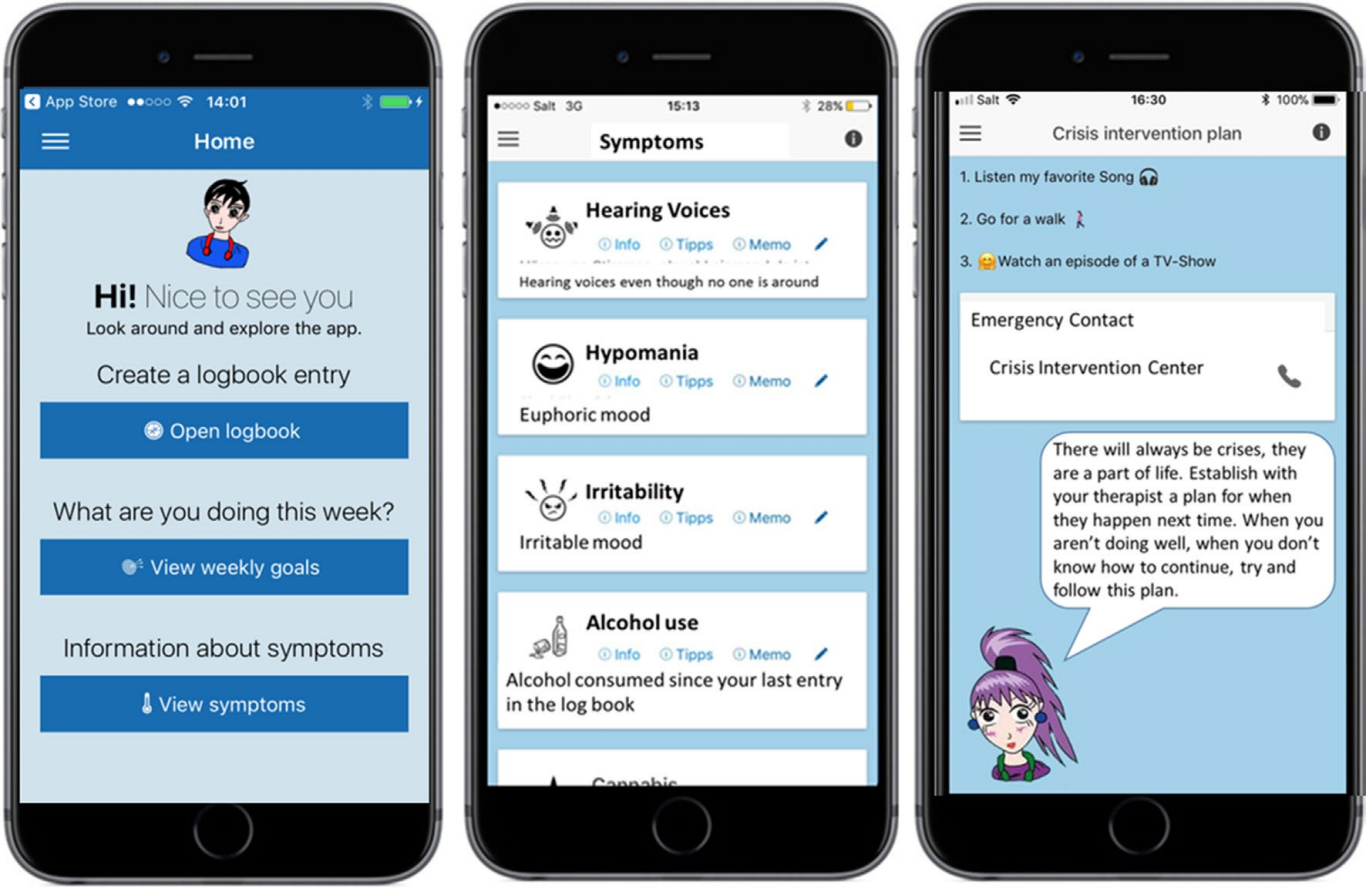
Example screenshots of the app Robin Z.

The app Robin Z (**Figure 2**) was developed to enrich the treatment sessions. A modular version of the app was developed and modified after the first pilot investigations with patients (*N* = 7, aged 14–18) and clinicians from the CAPS (*N* = 10). All clinicians within the pilot study reported that they would like to use the app Robin Z to enrich their therapeutic approaches. All patients in the pilot project used the app in their daily lives. Modules containing information about symptoms and coping strategies were used most frequently by patients. The findings were used to improve and adapt the application. In September 2017, the development of Robin Z was finalized and published on iTunes and Google Play. The technical aspects of Robin Z are regularly maintained without changes to the content.

The app is developed as an adjunct to the therapy and not a replacement for appropriate medical treatment and management. It is not at treatment itself, and it is not seen as a medical product according to the Swiss Medical Devices Ordinance Act. The authors recommend using the app in combination with a psychological treatment. Furthermore, it may be a used after completion of the study and/or therapy.

The app works offline and is not connected to the internet. Data collected by the app are stored in a private directory. These data are protected by the operating system and are inaccessible to other software on the device. The app does not transfer or store any data on the internet, and no data are stored outside of its private directory. In addition, the app is also password protected.

The app contains the following features:

**Symptom management:** Users will receive information about their symptoms and suggestions for managing them *via* the app. In therapy, these coping strategies are discussed with the patient and, if necessary, explained by the clinician. The adolescents can also define their own coping strategies and note helpful thoughts under the function “Memo.” The adolescents will be asked about their symptoms and their mood several times a day. They agree together with their therapists how many prompts they will receive each day. The symptom records are saved under a “Logbook.”

**Medication:** The individuals can record their medication, and they can set up a reminder. Medication adherence can be monitored through the Logbook.

**Crisis intervention:** In collaboration with their therapists, adolescents can set up a crisis intervention plan including emergency contacts. They can call the emergency contact directly *via* the app.

**Weekly goals:** The individuals can set their own goals. This means small, attainable goals so that they experience success. Furthermore, they are supported when dealing with their daily challenges. The goals are decided in the therapy session.

**Library:** The “Library” offers positive reinforcement for dealing with daily life. It consists of a list of strengths and positive experiences. The lists of strengths and positive experiences are discussed and extended on every week in therapy. There are suggestions for positive activities that the adolescents can engage in daily. The individuals are encouraged to do at least one positive activity per day. The suggestions are split into the following categories:

- Reward
- Creative activities
- Movement
- Social interaction
- Cognitive training
- Wellness
- Relaxation exercises

#### Standard Care

The control condition is composed of a control group from a former early recognition study in Zurich (ZInEP) [e.g., Ref. (73)]. The control group consists of 62 adolescents meeting CHR criteria (25 female, age range 13–18 years, mean age 15.06) who historically signed a declaration of consent regarding the use of their data. The sample was accrued from April 2010 to July 2012. The individuals of this subsample of the ZInEP had been treated with non-standardized psychological intervention (TAU) also at the CAPS. Within the ZInEP study, the comparable primary and secondary outcomes (at-risk symptoms, comorbid symptoms, global functioning, self-efficacy, and quality of life) have been evaluated at the same time points (at baseline and 6, 12, 24, and 36 months later). Since we expect a high interindividual variability of symptom fluctuation, reduction, or progression during the phase of intervention, we plan to reduce further putative impact of sex and age and to match individuals from the intervention group to ZInEP individuals accordingly. Consequently, 30 individuals from the ZInEP sample will be included in the statistical analysis.

### Primary Outcome

Primary outcome measures are transition to psychotic disorders, changes in at-risk as well as comorbid symptomatology, global functioning, life quality, and self-efficacy. All these measures will be compared with those of the control group.

### Secondary Outcome

Secondary outcome measures are the feasibility, user-friendliness, and acceptance of the treatment program Robin and the smartphone application Robin Z. Therefore, discontinuation of the therapy and subjective satisfaction with the therapy will be assessed. Furthermore, data about hospitalization, suicidality, and medication adherence will be collected and analyzed in comparison with those of the control group.

### Assessment

At baseline, demographic variables, past psychiatric illness, and treatment information are obtained from the participants and their caregivers. Before participating in the study, all patients will undergo a clinical assessment and diagnostic process according to *International Classification of Diseases*, Tenth Revision, criteria. Adolescents will be asked to complete a questionnaire about their personality traits PSSI (74) and to perform the Wechsler Intelligence Scale for Children (75) or Wechsler Adult Intelligence Scale for individuals aged > 16 years 11 months (76) to evaluate their cognitive profile.

In accordance with EPA guidance on the early detection of CHR states of psychoses, the CHR status is assessed with the Schizophrenia Proneness Interview Child and Youth Version (71) and the Structured Interview for Prodromal Syndromes (72). All investigators are either psychologists or psychiatrists. They are trained to a “gold standard,” and reliability will be measured for the duration of the trial. Comorbid mental disorders will be screened for with the Mini-International Neuropsychiatric Interview for Children and Adolescents based on *Diagnostic and Statistical Manual of Mental Disorders*, Fourth Edition (77). The severity of depression will be measured using theHamilton Depression Scale (78).

Information about functioning will be measured with the Global Assessment of Functioning Scale (GAF) (79), Social and Occupational Functioning Assessment (SOFA) Scale (80), and Global Functioning: Social Scale (GF: Social) (81). Data about quality of life, personality traits, and self-efficacy will be collected using the Manchester Short Assessment of Quality of Life (MANSA) (82) and the General Self-Efficacy Scale (SWE) (83).

Treatment satisfaction will be evaluated with the treatment evaluation questionnaire for adolescent patients FFB-J (84). A feedback form created specifically for the app will gather information regarding acceptance and contentment with the smartphone application Robin Z (**Table 1**).

**Table 1:** Description of assessments from baseline to endpoint of study.

	Baseline	Intervention (16 session)		Follow-up		
**Time point (months)**	**0**	**2**	**4**	**6**	**12**	**24**
Socio‐demographic characteristics	x	x	x	X	x	x
Clinical diagnosis according to**** *ICD-10* Re-evaluation of all diagnoses	x	x	x	X	x	x
Medication	x	x	x	X	x	x
Intellectual ability: WISC/WAIS	x					
Personality traits: PSSI	x					x
Clinical High Risk State: SPI-CY SIPS	x x	x	x x	x x	x x	x x
Comorbidities: MINI KID	x	x	x	x	x	x
Depression: HAMD	x	x	x	x	x	x
Functioning: GAF, GF: Social SOFAS	x x x	x x x	x x x	x x x	x x x	x x x
Quality of Life: MANSA	x	x	x	x	x	x
Self-efficacy: SWE	x	x	x	x	x	x
Satisfaction with treatment: FFE Feedback form regarding app Robin			x x			

GAF, Global Assessment of Functioning Scale; GF, Social, Global Functioning: Social Scale; HAMD, Hamilton Depression Rating Scale; ICD-10, International Classification of Diseases, Tenth Revision; MANSA, Manchester Short Assessment of Quality of Life; SIPS, Structured Interview for Prodromal Syndromes; SOFAS, Social and Occupational Functioning Assessment Scale; SWE, General Self-Efficacy Scale; WAIS, Wechsler Adult Intelligence Scale; WISC, Wechsler Intelligence Scale for Children.

### Statistical Analysis

Statistical analysis will be reported in accordance with the Consolidated Standards of Reporting Trials statement.

### Clinical Analyses

Differences in the primary and secondary outcomes between the two study groups at all time points will be examined with repeated measures analysis of variance and standardized effect sizes (Cohen’s *d*) will be reported. Their respective confidence intervals will be calculated for comparison. Response and remission rates will be compared across groups with contingency tables and differences tested with χ^2^. All unidirectional hypotheses will be tested one sided; bidirectional hypotheses will be tested two sided. Alpha is set to *p* < 0.05. All analyses will be conducted within SPSS.

A linear mixed model approach will be used to assess differences in the primary and secondary outcomes between the two study groups. Logistic regression will be used to assess response and remission rates across the groups. Medication, education, and intelligence will be taken into account as confounding factors by including them in mixed model and logistic regression model, respectively. All unidirectional hypotheses will be tested one sided; bidirectional hypotheses will be tested two sided. Significance level α is set to 0.05.

### Sample Size

The sample size calculation is based on testing differences between the TAU group and the intervention group. It has been assumed that the intervention group could achieve at least 10% additional improvement than the TAU group for the main instruments at the 6-month follow-up assessment (**Table 2**). Standard deviations for the different instruments have been estimated with data from adolescent participants of a previous early recognition study (73). Sample size calculation was assessed using SAS PROC Power, Version 9.4.

**Table 2:** Numbers needed to detect a significant difference between TAU and the intervention group for the different psychological instruments.

Instrument	Assumed additional improvement in comparison with TAU (∑ Total points per scale)	Maximum (∑ Total points)	*N* per group
SIPS	−15	114	17
SPI-CY	−9	84	16
SWE	+4	40	27
GAF	+10	100	24

GAF, Global Assessment of Functioning Scale; SIPS, Structured Interview for Prodromal Syndromes; SPI-CY, Schizophrenia Proneness Interview Child and Youth Version; SWE, General Self-Efficacy Scale; TAU, treatment as usual.

To the best of our knowledge, there is no comparable study reporting effect sizes of the improvement of clinical outcome variables such as overall symptom dimensions and functioning in CHR adolescents. Considering the respective outcome measures, the sample size ranges from 16 to 27 (**Table 2**). In a conservative approach, we chose to include 30 individuals per group.

### Data Monitoring and Management

Study data will be entered onto a study specific database (Research Electronic Data Capture REDCap) following a standard operating procedure. All data will be collected on paper case report forms (CRFs) that are anonymized and stored at the CAPS. Data will be entered by researchers electronically into the REDCap database. A random subset of data will be checked for quality control by independently checking the paper CRFs, and electronically entered data and any errors will be corrected. Only the leading investigators, trial statistician, and researchers will have access to the final dataset.

Serious adverse events are regularly monitored and documented by the research team and reported immediately to the chief investigator and/or a senior clinical member of the team.

### Ethical Issues

This study is being conducted in accordance with globally accepted standards of good clinical practice, in agreement with the Declaration of Helsinki and with national regulations of the Swiss Ethics Committees on research involving humans. The local ethics committee in Zurich has approved the study procedure, study information, and informed consent forms in Mai 2017 before the inclusion of a first patient in the trial. The study has been registered at the international trial register ClinicalTrials.gov (ClinicalTrials.gov identifier NCT03829527) and the national trial register kofam (kofam.ch identifier SNCTP000003148).

Every participant can drop out of the study at any time. All dropouts will be documented in the CRFs. The study investigators ensure that all mental health professionals involved in the study are qualified and informed about the protocol, interventions, and trial-related duties.

## Discussion

Despite evidence suggesting that young individuals with at-risk symptoms may profit best from specialized treatment approaches (85), little is known about age-appropriate treatment strategies in this vulnerable age group (15). The treatment program Robin has been developed to fill this gap.

Our treatment approach Robin is an integrated approach based not only on CBT but also on systemic therapy. The treatment is both solution and resource focused. Furthermore, Robin is an individualized treatment approach in line with the individual needs of the particular patients, which should lead to a high acceptance. Furthermore, age-appropriated treatment material, including a smartphone app and the involvement of the family, may result in higher engagement in therapy (32, 41, 49). The smartphone application Robin Z has been developed to enrich the treatment and to increase treatment satisfaction through providing support between sessions. Previous studies have already demonstrated that mobile-based interventions are feasible and accepted by psychotic patients (49, 58, 59, 66); thus, we expect the same for CHR individuals.

In accordance with EPA guidance in early intervention in CHR states of psychoses (15), a primary aim of this treatment approach is to improve the daily functioning. Thus, the study group decided to primarily focus on quality of life, self-efficacy, and functioning. These areas have been shown to be most impacted in adolescents and often prompt them to seek help (18, 30, 44, 86, 87). In contrast, at-risk symptoms are not necessarily the cause of the highest psychological strain and burden for the patient. Furthermore, we expect a remission or at least a significant decline of the at-risk symptoms in most patients after the treatment period. Prior evidence has demonstrated that at-risk symptoms, especially in adolescents, show high levels of fluctuation (70, 88). How much this fluctuation symptoms or remission rates are associated with a specific intervention is currently unknown. Our first analysis of symptomatology in the control group also showed a high reduction of at-risk symptoms during follow-up compared with the baseline.

The integrated and age-adapted treatment approach as well as the focus on outcomes other than the putative progression of symptomatology and onset of psychosis also seems to be supported by a recent network meta-analysis. Davies et al. (89) compared efficacy of different preventive interventions in CHR individuals considering conversion rates in psychosis and acceptability of treatment intervention. The findings demonstrated a lack of evidence to favor a specific intervention to prevent onset of psychosis, and there were no significant differences in acceptability between the treatment approaches. In the context of non-superiority of any intervention, the authors questioned CBT as the most widely adopted intervention in the early intervention of psychosis.

Using a control group of our previous early recognition study ZInEP and a non‐randomized design could be possible sources of bias. However, data of the control group participants of ZInEP were reassessed at the same time points (6, 12, and 24 months), and identical measures were used. The TAU was carried out in the same outpatient service under the supervision of M. F. There were no major changes in the Swiss health system between 2010 and 2018 that could have had an influence on the frequency or intensity of therapeutic sessions. The outpatient mental healthcare system in Zurich has remained widely unchanged since then. Another limitation of the control group may be the naturalistic setting. As their treatment was not standardized, many treatment variables, such as the treatment approach or the therapists themselves, cannot be controlled for. However, even though a moderate sample size may limit statistical controlling, important confounders such as effect of medication, hospitalization, and treatment frequency may be identified in both groups and controlled for. Our control group is an extensively and carefully assessed large sample of CHR adolescents (*N* = 62) in the same age range and from the same geographical area. The sample is double that of the intervention sample and allows for age and sex matching to individuals in the intervention study. Consequently, more resources can be allocated to recruiting the experimental group, and therefore, the chance of achieving a larger sample will be increased. This will also help to reduce ethical concerns, as the help-seeking patients will be reporting a high level of distress and requesting a specific treatment. The treatment is based on the current research results in the field of early intervention and prevention of psychosis, as well on the clinical expert knowledge from the therapists in the early intervention center. Therefore, our study team will act on the assumption that the treatment approach Robin will lead to the best possible outcome.

Another reason for utilizing a control group from a former study is the smartphone application Robin Z. Since the application can be downloaded for free from App Store and Google Play, it seems difficult to eliminate Robin Z as a confounder. Even if the therapists do not ask the control participants to download the application, their use of it cannot be ruled out. Although the usage of Robin Z alone will not replace treatment, the authors assume that combination of psychological intervention and the use of the app will lead to a better psychological outcome.

To the authors’ knowledge, this is the first controlled trial to test the efficacy of a specific treatment manual in combination with a smartphone application for adolescent patients at CHR. The results of the study are expected to add important knowledge within the research fields of prevention and early intervention in psychosis in adolescents within the peak age of psychosis onset.

## Dissemination

After study completion, the results of the primary and secondary analyses will be published in international peer-reviewed journals (at least one specialized in addiction and another in adolescent psychiatry).

## Trial Status

The recruitment started in September 2017. Participants will be included till September 2020.

## Ethics Statement

This study was carried out in accordance with the recommendations of Swiss Ethics Committees on research involving humans with written informed consent from all subjects. All subjects gave written informed consent in accordance with the Declaration of Helsinki. The protocol was approved by the local Ethic committee in Zurich.

## Author Contributions

NT-W, SW, and MF designed the present study ETRo. NT-W, MG, SM, MJ, MK, NS, MZ-L, SW, and MF contributed to the development of the treatment manual and the smartphone application Robin for the intervention group. NT-W, MG, SM, and MF are collecting the data. AR is the data analyst for the project and undertook the statistical analysis. WR and SW designed the study of ZInEP “The Zurich Early Recognition Program” for the control group. NT-W, MG, SM, and MF collected the data for the control group. All authors have contributed to and have approved the final manuscript.

## Conflict of Interest Statement

SW has received in the last 5 years royalties from Thieme, Hogrefe, Kohlhammer, Springer, and Beltz. Her work was supported in the last 5 years by the Swiss National Science Foundation (SNF), diff. EU FP7s, HSM Hochspezialisierte Medizin of the Kanton Zurich, Switzerland, Bfarm Germany, ZInEP, Hartmann Müller Stiftung, Olga Mayenfisch, and Gertrud Thalmann Fonds. Outside professional activities and interests are declared under the link of the University of Zurich, www.uzh.ch/prof/ssl-dir/interessenbindungen/client/web/.

The remaining authors declare that the research was conducted in the absence of any commercial or financial relationships that could be construed as a potential conflict of interest.
